# Increasing age of multiple sclerosis onset from 1920 to 2022: a population-based study

**DOI:** 10.1007/s00415-023-12047-9

**Published:** 2023-12-14

**Authors:** A. Habbestad, J. S. Willumsen, J. H. Aarseth, N. Grytten, R. Midgard, S. Wergeland, K. M. Myhr, Ø. Torkildsen

**Affiliations:** 1 Neuro-SysMed, Department of Neurology, Haukeland University Hospital, 5021 Bergen, Norway; 2 Department of Clinical Medicine, University of Bergen, Bergen, Norway; 3 Norwegian Multiple Sclerosis Registry and Biobank, Haukeland University Hospital, Bergen, Norway; 4 Department of Neurology, Molde Hospital, Molde, Norway; 5 Department of Neuromedicine and Movement Science, Norwegian University of Science and Technology, Trondheim, Norway

**Keywords:** Multiple sclerosis, Age, Sex-difference, Prevalence, Cohort

## Abstract

**Objective:**

To study the age at onset of relapsing–remitting multiple sclerosis (RRMS) during the past century.

**Methods:**

This is a population-based cohort study of persons diagnosed with RRMS in Hordaland, Møre, and Romsdal counties, Western Norway, from 1920 to 2022. Individual patient data were available and assessed from previously conducted prevalence and incidence studies in addition to hospital records up until October 31, 2022. Participants were categorized according to onset period and analyzed for temporal trends in age at onset, time from onset to diagnosis, and distribution of onset over time.

**Results:**

We identified 3364 persons with confirmed RRMS. The mean age at onset significantly increased (*p* < 0.001) throughout the study period, despite a decrease in time from symptom onset to diagnosis (*p* < 0.001). The proportion of persons with MS onset after 50 years of age increased from 2.6% before 1970 to 11.9% after 2010**.** We also found a trend toward a bimodal distribution of age at onset that peaked at around 30 years and 40–45 years of age in the latest period.

**Conclusion:**

Age at onset of MS significantly increased throughout the study period. This was mainly due to an increasing number of persons with MS, predominantly female, experiencing onset after 40–45 years of age. This bimodal distribution could indicate different susceptibility periods of MS or changes in exposure to risk factors during the observation period*.*

**Supplementary Information:**

The online version contains supplementary material available at 10.1007/s00415-023-12047-9.

## Background

Multiple sclerosis (MS) is a chronic inflammatory and degenerative disease of the central nervous system. The two main onset types are relapsing–remitting MS (RRMS) and primary progressive MS (PPMS) [1]. The disease typically manifests in young adults between 20 and 40 years of age and often causes significant disability over time [1]. Several studies, both in Norway and in other countries, report an increasing incidence of MS [2–17]. Western Norway has one of the highest reported prevalence rates, with a prevalence ratio of 335.8 per 100.000 inhabitants, and an incidence ratio of 14.4 per 100.000 in Møre and Romsdal counties [12].

Adult-onset MS (AOMS) is often defined as MS onset between 18 and 40–50 years of age [18, 19]. Early (juvenile) onset MS (EOMS) is usually defined as onset before 16–18 years of age, whereas late-onset MS (LOMS) implies MS onset after 50 years of age [19, 20]. Increasing incidence of MS onset above the age of 40–45 years has recently been reported in different populations with a high rate of LOMS [21–24]. Furthermore, there has also been reported an increasing incidence of MS among female, most pronounced with late onset [22]. Whether these observed changes represent a global trend is unknown.

The aim of this study was to investigate whether age at onset changed from 1920 to 2022 in a well-defined population of persons with MS (pwMS).

## Methods

### Study design

This population-based cohort study comprised Hordaland, Møre, and Romsdal counties in Western Norway. The MS epidemiology has previously been thoroughly studied in both counties [2–12]. The healthcare system in Norway is mainly public, meaning that every citizen is universally covered by the welfare system, ensuring equal access to healthcare services for all citizens. Thus, all persons with suspected MS are referred to public hospitals for diagnosis and treatment. Throughout the study period, the few private practice neurologists in the counties have all referred cases of suspected MS to the Departments of Neurology at the local public hospitals.

The participants in the present study were diagnosed with MS according to the diagnostic criteria during the study period [25–29]. These criteria have undergone several revisions over time, initially based on neurological symptoms and clinical findings only, to more recent diagnostic criteria including neurophysiological examinations, cerebrospinal fluid analysis, and magnetic resonance imaging (MRI), making the diagnostic process faster and more accurate.

### Data collection and study population

Data files with individual data were available from previously conducted prevalence and incidence studies in Hordaland, Møre, and Romsdal counties from 1953 to 2013 [2–12]. Persons diagnosed after 2013 were identified from the patient records in the Department of Neurology at Haukeland University Hospital, Bergen, and in the Department of Neurology, Molde Hospital, Møre, and Romsdal. All patient records were reassessed according to time of diagnosis and place of residence up until October 31, 2022. The Department of Neurology at Haukeland University Hospital was established in 1953 and is responsible for the neurological health service to residents in central and northern regions of Hordaland County, while the Department of Neurology at Molde hospital was established in 1960 and is together with an outpatient clinic in Ålesund responsible for all pwMS in Møre and Romsdal counties. All persons with MS in Hordaland, Møre, and Romsdal counties were referred from general practice and private neurological practices to these hospitals for diagnostic evaluation, including MRI and diagnostic cerebrospinal fluid (CSF) analysis. Thus, all persons diagnosed with MS in these two counties are therefore most likely included in the sample.

**Fig. 1:**
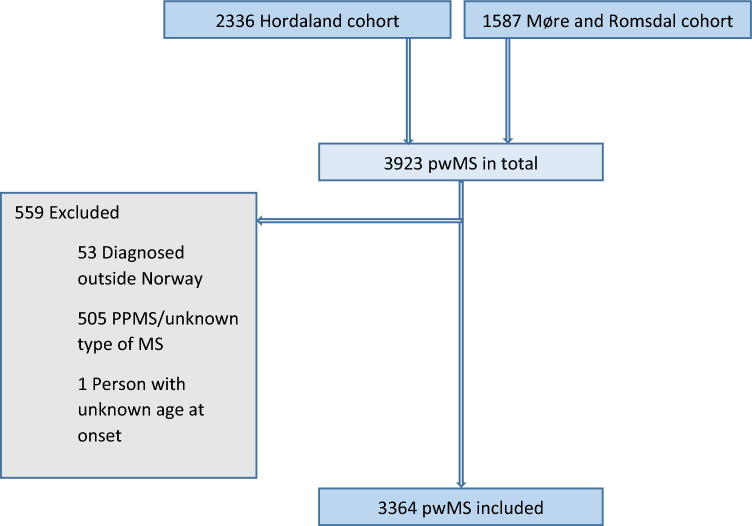
Flowchart of the study population of persons with multiple sclerosis in Hordaland, Møre, and Romsdal counties, Western Norway

The following data were collected for all participants: year of birth, sex, year of onset (first clinical event suggestive of MS reported by participants or relatives), and year of diagnosis and MS phenotype at onset (relapsing or progressive). All Norwegian inhabitants have a unique personal identification number, ensuring that there were no duplicates in our cohort. After identification, all data were anonymized and stored in accordance with the EU 2016/679 GDPR. We defined onset as the calendar year in which participants recalled their first symptom suggestive of MS. Since the time of onset of PPMS is often more uncertain than RRMS, persons with PPMS were excluded from the primary analyses. To avoid an effect of differences in migration patterns over time, we also excluded persons diagnosed with MS outside of Norway (Fig. 1).

### Statistical methods

The study populations from the two counties were merged into one cohort. Participants were categorized according to onset period: < 1970, 1970–1979, 1980–1989, 1990–1999, 2000–2009, and 2010–2022. We analyzed the data for mean (± SD) age at onset and the interval from onset to diagnosis for each period separately. We also estimated the distribution of onset over time using a density plot. The temporal trend was analyzed by the Jonckheere–Terpstra test, and the Cochran–Armitage test for trend was used to test for change in female-to-male ratio over time. Finally, we calculated the female-to-male ratio for all pwMS with disease onset during these time periods and across four different age groups (< = 25, 26–35, 36–45 and > 45)**.** Statistical analyses were performed in R version 4.2.2

### Standard protocol approvals

The study was approved by the Regional Committee for Medical and Health Ethics in Western Norway.

## Results

We identified 3923 pwMS, of whom 599 were excluded. Fifty-three were excluded because they were diagnosed outside of Norway and one participant because of unknown age at disease onset. Of the remaining 3869 participants, we excluded 505 (253 females and 252 males, 13.1%) with PPMS or unknown disease course. The mean (± SD) age at onset for these excluded persons was 42.45 (± 11.9), four (0.8%) of them had an EOMS (before 18 years), while 142 (28.1%) had LOMS (after 50 years). Data on both RRMS and PPMS combined are given in eTable 1 and eFigures 1–2).

**Fig. 2:**
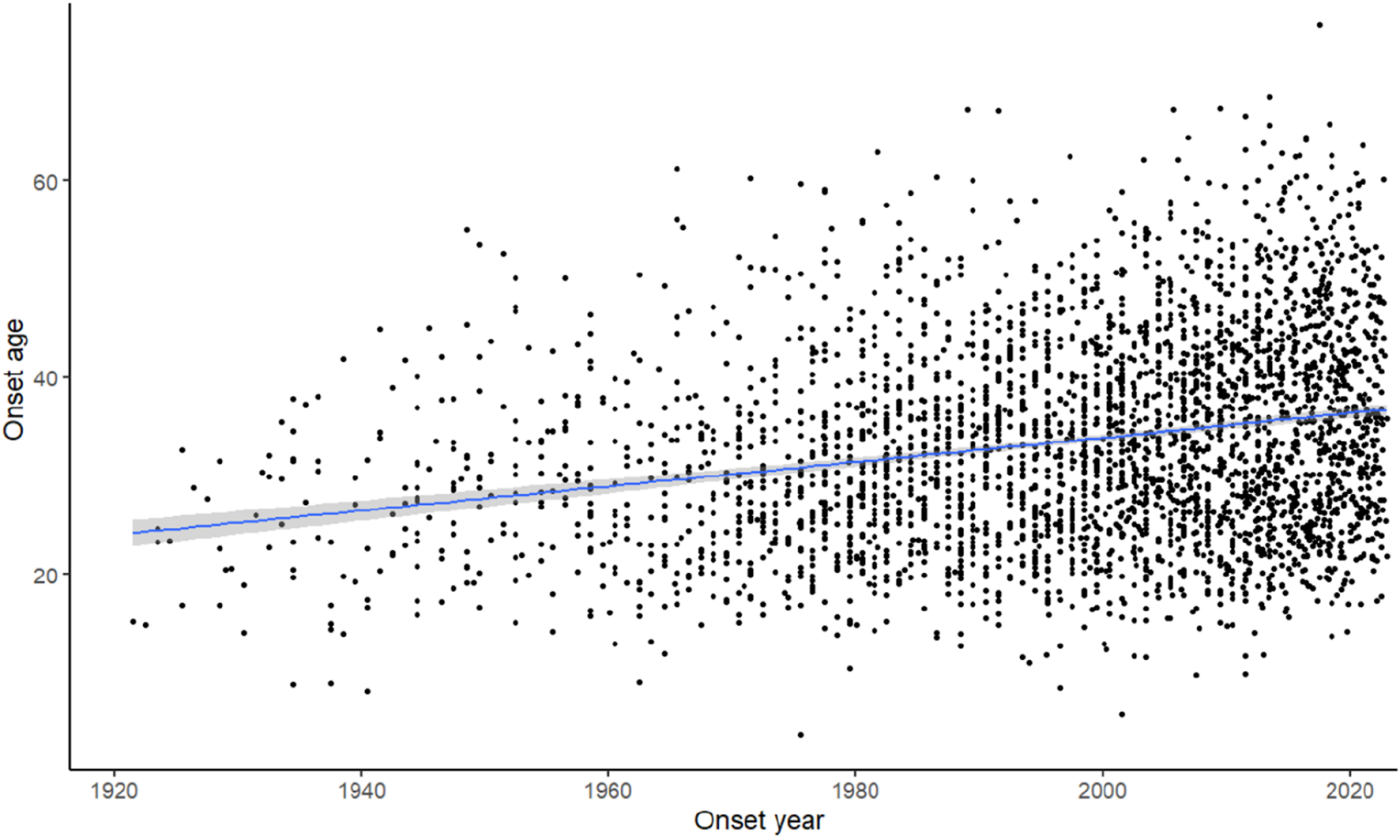
Age and year at onset of relapsing–remitting multiple sclerosis

The main analysis included 3364 participants with RRMS, including 1107 (32.9%) male and 2257 (67.1%) female, with MS onset between 1920 and 2022 (Table 1, Fig. 2). The mean (SD) age at onset was 33.5 ± 10.4 years, ranging from 4 to 76 years, and the mean (SD) interval from symptom onset to diagnosis, i.e., the diagnostic delay, was 4.5 (± 6.9) years, ranging from 0 to 59 years.

**Table 1:** Number of persons with relapsing–remitting multiple sclerosis and age at onset in different decades

Disease onset	Male		Female		Total	
	*n*	Mean (SD)	*n*	Mean (SD)	*n*	Mean (SD)
< 1970	147	30.7 (9.4)	233	27.3 (8.5)	380	28.6 (9.0)
1970–1979	97	31.7 (9.8)	214	30.4 (9.6)	311	30.8 (9.7)
1980–1989	162	32.5 (10.4)	277	32.3 (9.0)	439	32.4 (9.5)
1990–1999	164	33.1 (9.5)	366	32.9 (9.8)	530	32.9 (9.7)
2000–2009	232	34.6 (10.5)	497	34.9 (10.2)	729	34.8 (10.3)
2010–2022	305	35.2 (10.8)	670	36.6 (11.2)	975	36.2 (11.1)
Total	1107	32.9 (10.3)	2257	33.5 (10.5)	3364	33.5 (10.4)

**Fig. 3:**
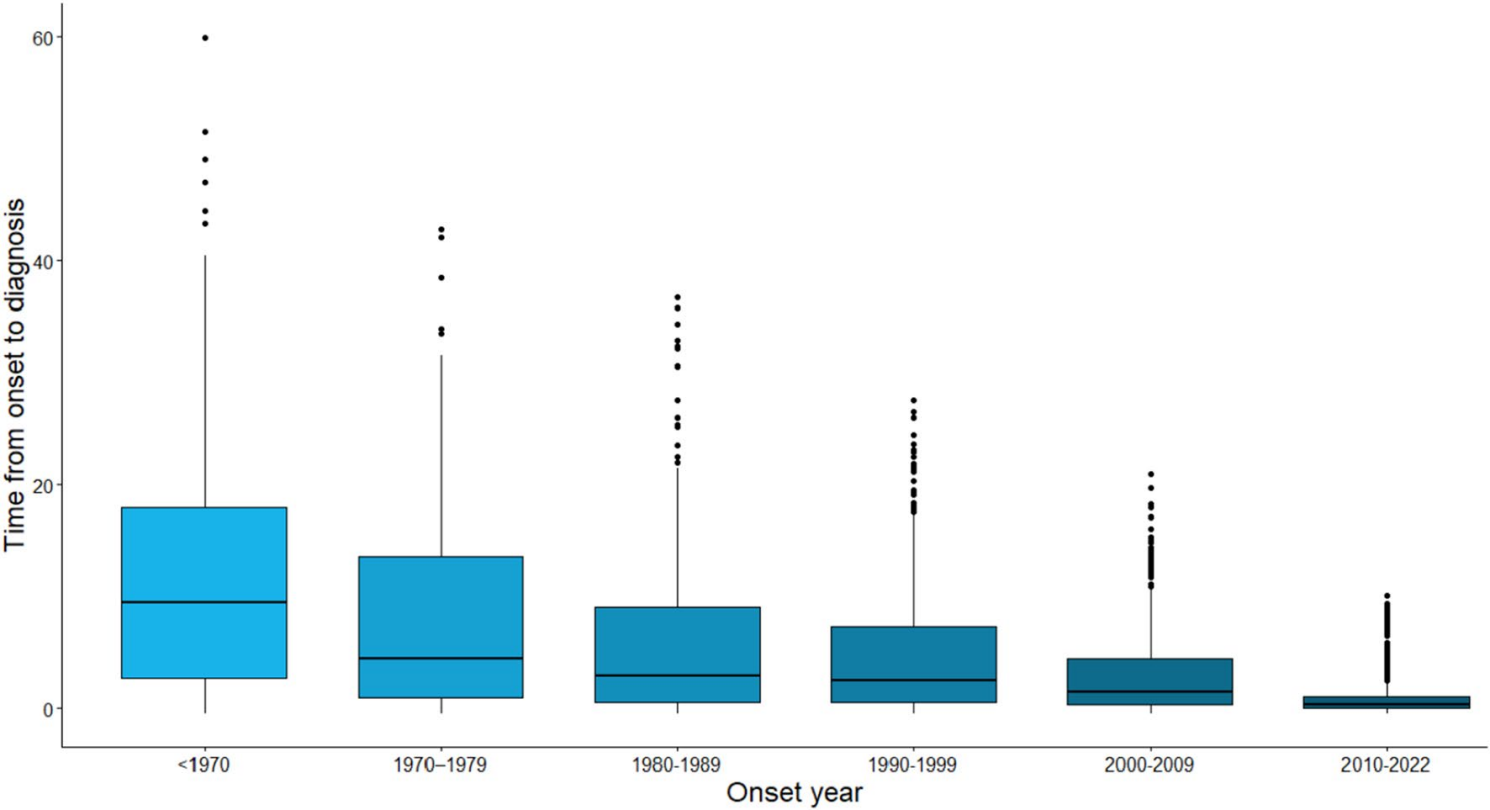
Time from onset to diagnosis of multiple sclerosis stratified for decades of onset symptoms

There was a significant increase in mean age at onset during the study period (*p* < 0.001) (Fig. 3a). The increase in mean age was caused by more pwMS diagnosed above the age of 40 years and not by fewer pwMS diagnosed in the younger age groups. The proportion of late-onset RRMS (after 50 years) increased from 2.6% before 1970 to 11.9% after 2010. We performed an additional sensitivity analysis including persons with PPMS that did not alter the results (eFig. 1–2).

### Time from symptom onset to diagnosis

**Fig. 4:**
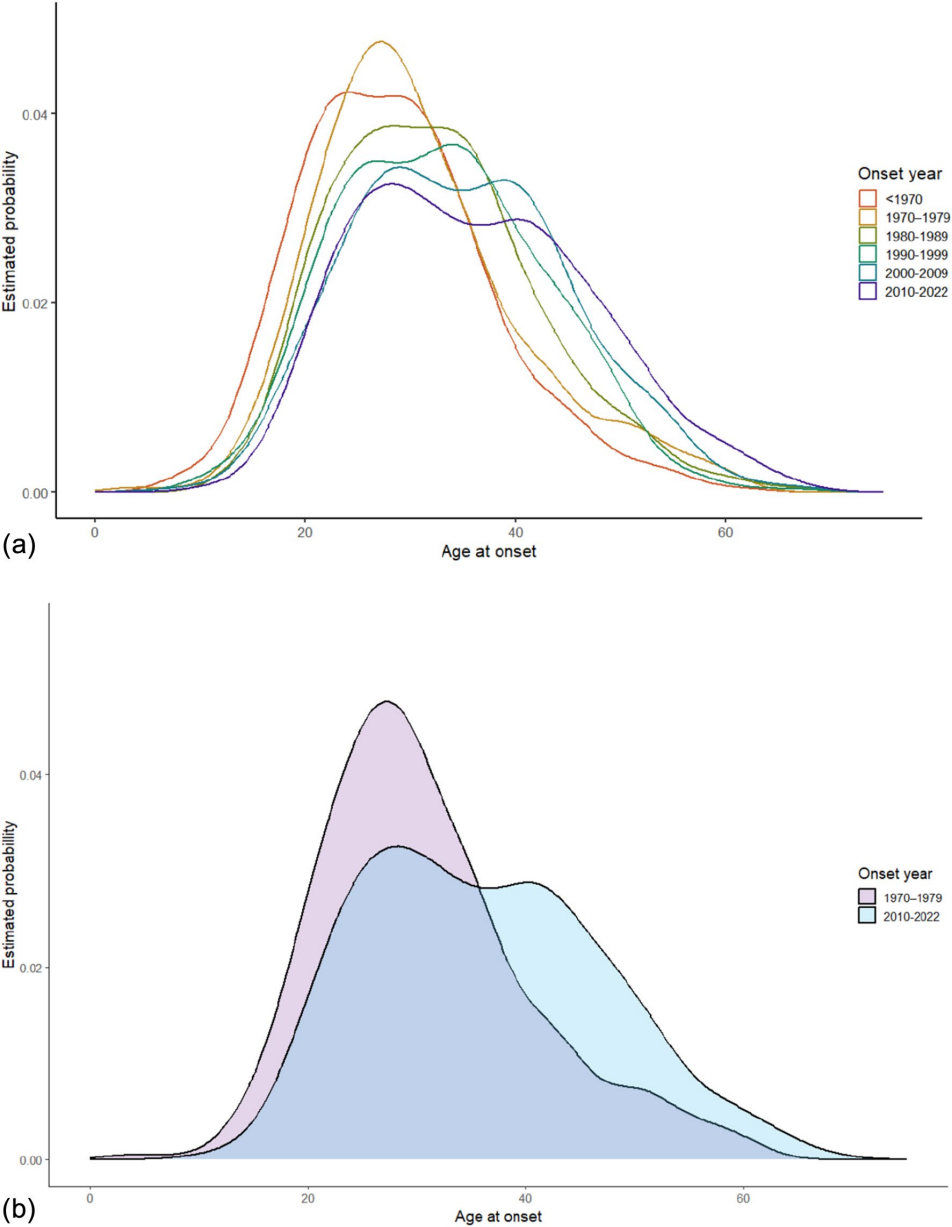
**a** Distribution of age at onset of multiple sclerosis for the entire study period. **b** Distribution of age at onset of multiples sclerosis during 1970–1979 compared to 2010–2022

The mean interval from symptom onset to diagnosis decreased over time, ranging from 11.7 (± 10.6) in the years before 1970 to 1.0 (± 1.7) during 2010–2022 (*p* < 0.001), (Fig. 4). Thus, we observed a profound decrease in diagnostic delay during the study period.

### Sex ratio

Female-to-male ratio remained around 2:1 for most of the period (eFig. 3). Excluding data from before 1970, which could suffer from less reliable person identification than the rest of the study period, we found no trend (*p* = 0.18). When analyzing sex ratio across age at onset, we found an increased F:M ratio in persons with onset > 45 years over time (*p* < 0.01).

### Density plots and age at onset distribution

We used a density plot to investigate the age at onset of MS in different decades. Figure 3a shows a density plot for the entire study period, while Fig. 3b compares the densities during 1970–1979 to 2010–2022. There was an increased probability of onset above 40 years during 2010–2022 compared to 1970–1979 (Fig. 3b). Furthermore, in addition to a shift to the right (increased age at onset), we also found a bimodal distribution of age at onset that peaked at around 30 years and 40–45 years of age (Fig. 3a). When comparing the density plots for male and female, the observed second peak at around 45 years of age appeared more prominent in female (eFigs. 4–5).

## Discussion

We found an increased age at onset of Norwegian pwMS over the past century, despite a significantly decreased interval from disease onset to diagnosis during the same period. This was mainly caused by an increasing number of pwMS, predominantly female, experiencing onset after 40 years of age.

Our findings are mostly consistent with previous reports, demonstrating increasing age at onset in several populations [13–15, 21–23]. A recent study from Italy found an increasing age at onset over time, a shortened interval from onset to diagnosis, and an increased female-to-male ratio in older age groups [21]. A large, population-based study from Denmark also reported an increased incidence in female with MS, particularly among those older than 50 years of age [22]. Further, a study from Spain reported an increasing age at onset over time [23]. Thus, our findings add to, and strengthen, the already reported increase in mean age at MS onset observed across different populations, supporting the validity of these findings. Previously published epidemiological studies in Norway are also consistent with these findings [13–15]. Although none of these studies have focused purely on this topic, some of them have reported a slight increase in age at MS onset and an increased proportion of pwMS experiencing onset at an older age.

We did not only observe an increase in average age at onset, but also the distribution of age at onset has changed over time. Other studies have not highlighted this latter observation, as illustrated by the density plots [21, 23]. While a large proportion of pwMS in the present study experienced disease onset during their early adult life, we also observed a higher proportion of participants with LOMS. The density plot from the different decades showed a trend toward a shift from a unimodal onset distribution peaking at about 30 years to a bimodal distribution at about 30 years and 40–45 years of age. This was most evident when comparing pwMS with onset during 1970–1979 with those with onset during 2010–2022. Sex-specific analyses suggest that this change was primarily driven by the high proportion of female with LOMS.

Several possible reasons may be associated with our findings. Previous studies have suggested changes in environmental factors, i.e., Epstein–Barr virus infections, vitamin D levels, smoking habits, or hormonal changes [21–23]. Another interesting observation is that the maternal age at first childbirth in Norway has increased from 24.6 years in 1961 to 30.1 years in 2021 [30], a change that has occurred in parallel with our observation of increasing age at MS onset. This is particularly interesting because both our study and other reports have demonstrated a disproportionally large proportion of female with LOMS in recent years, and sex-specific exposure may therefore be of interest. During the follow-up period, there has been a change in diagnostic criteria for MS [25–29]. This could potentially have affected our findings. On the one hand, new diagnostic criteria have allowed earlier and more accurate diagnosis (shorter time from onset to diagnosis, and larger number of people with formal diagnosis of MS, as shown in Fig. 3). On the other hand, there is greater attention to late-onset MS (second peak of MS diagnosis after the age of 40). Our results are in accordance with recent prevalence and incidence studies, confirming a bimodal peak, corresponding to 25–34 and 45–54 age groups [24].

The strength of this study is the long observation period of more than 100 years, and the relatively large population-based study sample is well-characterized through several previous epidemiological studies from Hordaland, Møre, and Romsdal counties [2–12]*.* Furthermore, Norway with a high prevalence of MS, combined with robust and equally distributed public health services and population-based health registries, is well suited for longitudinal studies on MS onset. Thus, we consider the study population as close to complete, due to the unique public healthcare system in Norway with no private clinics of importance in the area, combined with the national unique personal identification numbers that exclude duplicates.

Study limitations include the obvious concern of recall bias related to year of symptom onset, as time from symptom onset to diagnosis can take years, thus making the time of onset prone to bias. It may be difficult to identify early symptoms, and some participants in our study could therefore have a false late recorded onset. This would presumably be most pronounced among pwMS identified during the first part of the study period (before 1970) compared to later periods (after 2000) because of longer diagnostic delay. Secondly, the change in the diagnostic criteria for MS during the study period may also influence the study population. The healthcare system has also changed over the same period. Thus, the participants in our study have been diagnosed with MS according to different diagnostic criteria. The earliest criteria were clinically based and restricted MS onset between the age of 10 and 50 years [28, 29]. This could mean that some cases, especially in the older age groups, remained unidentified due to the age restrictions in the earlier revisions of the diagnostic criteria. It is also a concern that the number of pwMS diagnosed before 1970 was rather small. For this reason, we chose to merge all these participants into one group, and this could have made it difficult to detect actual changes taking place during that period. It is also important to address that our findings are applicable to Norway and not necessarily elsewhere or in other population groups. Nevertheless, our findings were consistent with the findings from previously studies [14, 21–23], indicating that there might have been similar changes in environmental risk factors in the different populations.

Our observation of the increasing proportion of participants with LOMS and the bimodal distribution of age at onset suggest changes in risk factor exposures. These findings require further studies of temporal changes in environmental factors and should lead to further studies on the age impact on the efficacy and adverse events of disease-modifying therapies.

### Supplementary Information

Below is the link to the electronic supplementary material.

Supplementary file1 (DOCX 602 KB)
