# Supplementary material for: Increasing age of multiple sclerosis onset from 1920 to 2022: a population-based study
Source: J Neurol. 2023 Dec 14;271(4):1610–7. doi: 10.1007/s00415-023-12047-9 (PMC10973050; doi:10.1007/s00415-023-12047-9)

**eSupplement**

eTable 1 Number of persons with relapsing-remitting and primary progressive multiple sclerosis combined, and age at onset in different decades.

eFigure 1 Age at onset of relapsing-remitting and primary progressive multiple sclerosis patients.

eFigure 2 Distribution of age at onset of multiple sclerosis for the entire study population, including both relapsing-remitting and primary

progressive multiple sclerosis patients.

eFigure 3 Female-to male sex ratio over time.

eFigure 4 Distribution of age at onset of relapsing-remitting multiples sclerosis during 1970-1979 compared to 2010-2022, men only.

eFigure 5 Distribution of age at onset of relapsing-remitting multiples sclerosis during 1970-1979 compared to 2010-2022, women only.

**eTable 1.** Number of persons with relapsing-remitting and primary progressive multiple sclerosis combined, and age at onset in different decades.

|  | **Male** | | **Female** | | **Total** | |
| --- | --- | --- | --- | --- | --- | --- |
| **Disease onset** | **n** | **Mean (SD)** | **n** | **Mean (SD)** | **n** | **Mean (SD)** |
| **<1970** | 192 | 32.1 (9.8) | 284 | 28.8 (9.4) | 476 | 30.1 (9.7) |
| **1970-1979** | 126 | 33.0 (10.1) | 251 | 31.2 (10.1) | 377 | 31.8 (10.1) |
| **1980-1989** | 196 | 33.7 (10.9) | 304 | 32.7 (9.2) | 500 | 33.1 (9.9) |
| **1990-1999** | 217 | 35.7 (10.3) | 419 | 34.2 (10.5) | 636 | 34.7 (10.5) |
| **2000-2009** | 286 | 37.1 (11.8) | 537 | 35.7 (10.7) | 823 | 36.2 (11.1) |
| **2010-2022** | 342 | 37.0 (12.1) | 715 | 37.5 (11.7) | 1057 | 37.3 (11.8) |
| **Total** | 1359 | 35.3 (11.2) | 2510 | 34.4 (11.0) | 3869 | 34.7 (11.1) |

**eFigure 1.** Age at onset of relapsing-remitting and primary progressive multiple sclerosis patients.


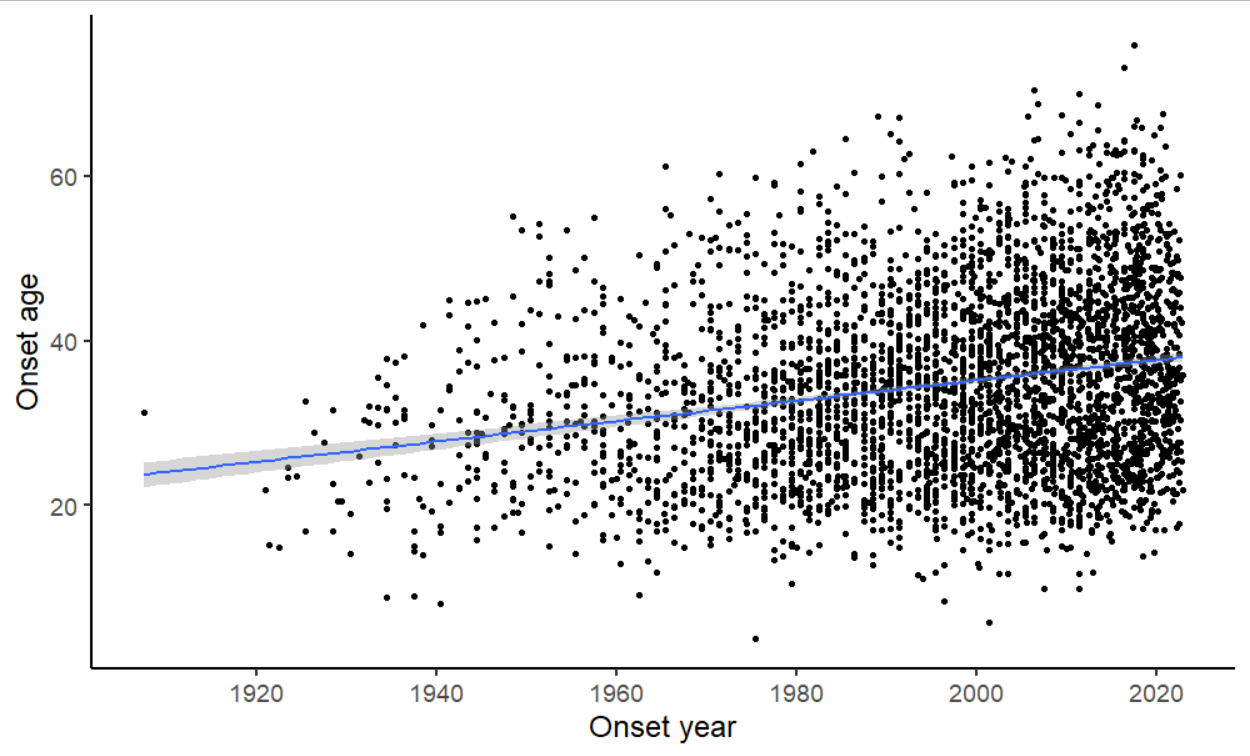
**eFigure 2.** Distribution of age at onset of multiple sclerosis for the entire study population, including both relapsing-remitting and primary progressive multiple sclerosis patients.


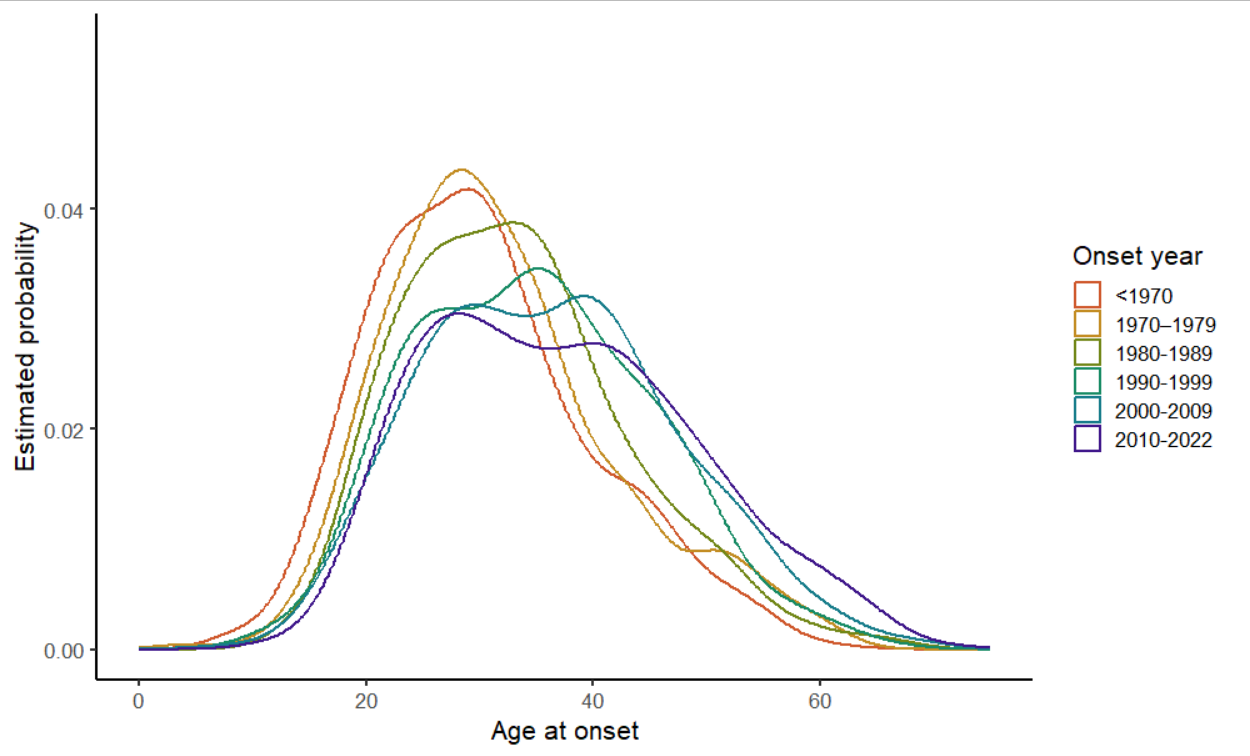


**eFigure 3**. Female-to male sex ratio over time.
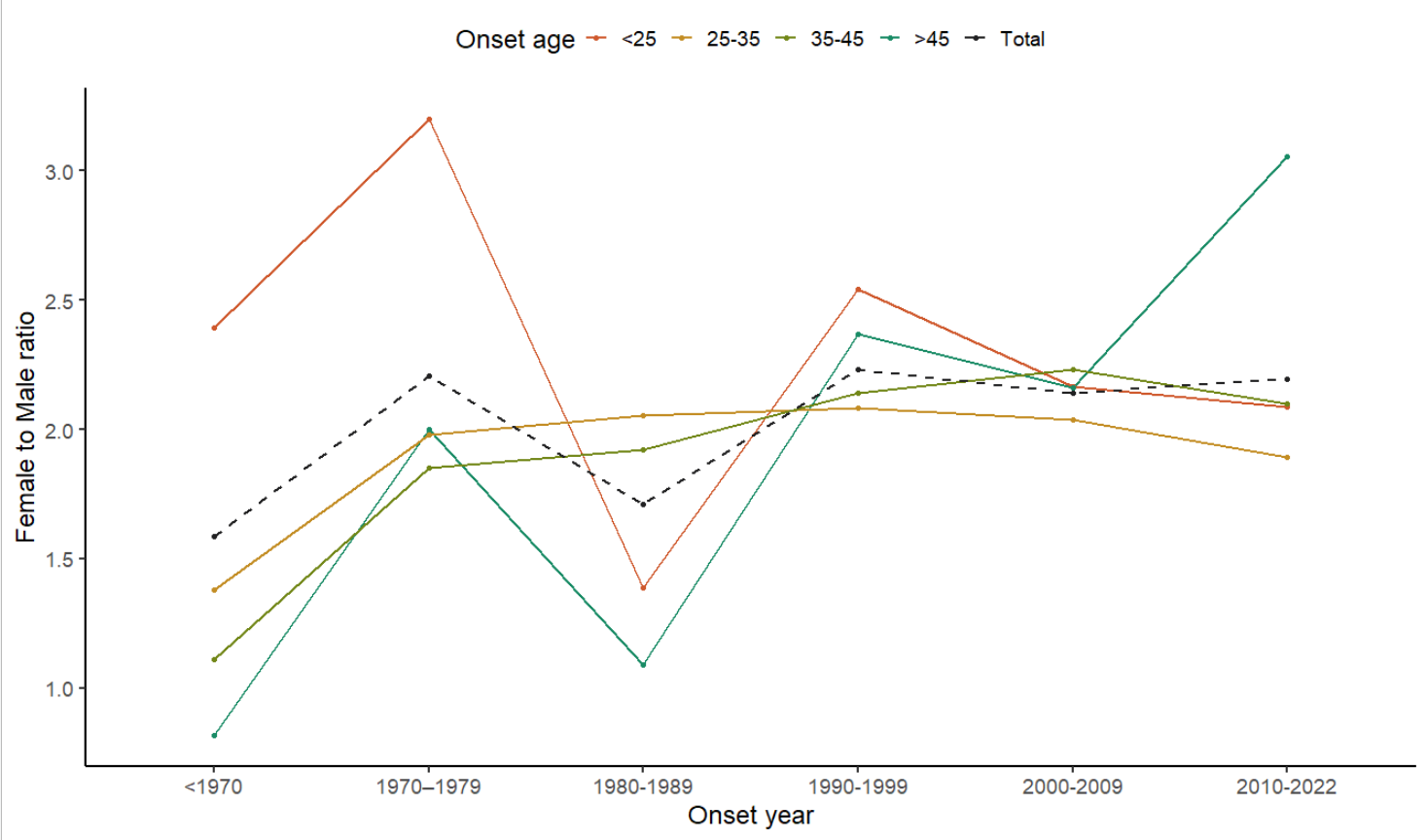


**eFigure 4**. Distribution of age at onset of relapsing-remitting multiples sclerosis during 1970-1979 compared to 2010-2022, men only.


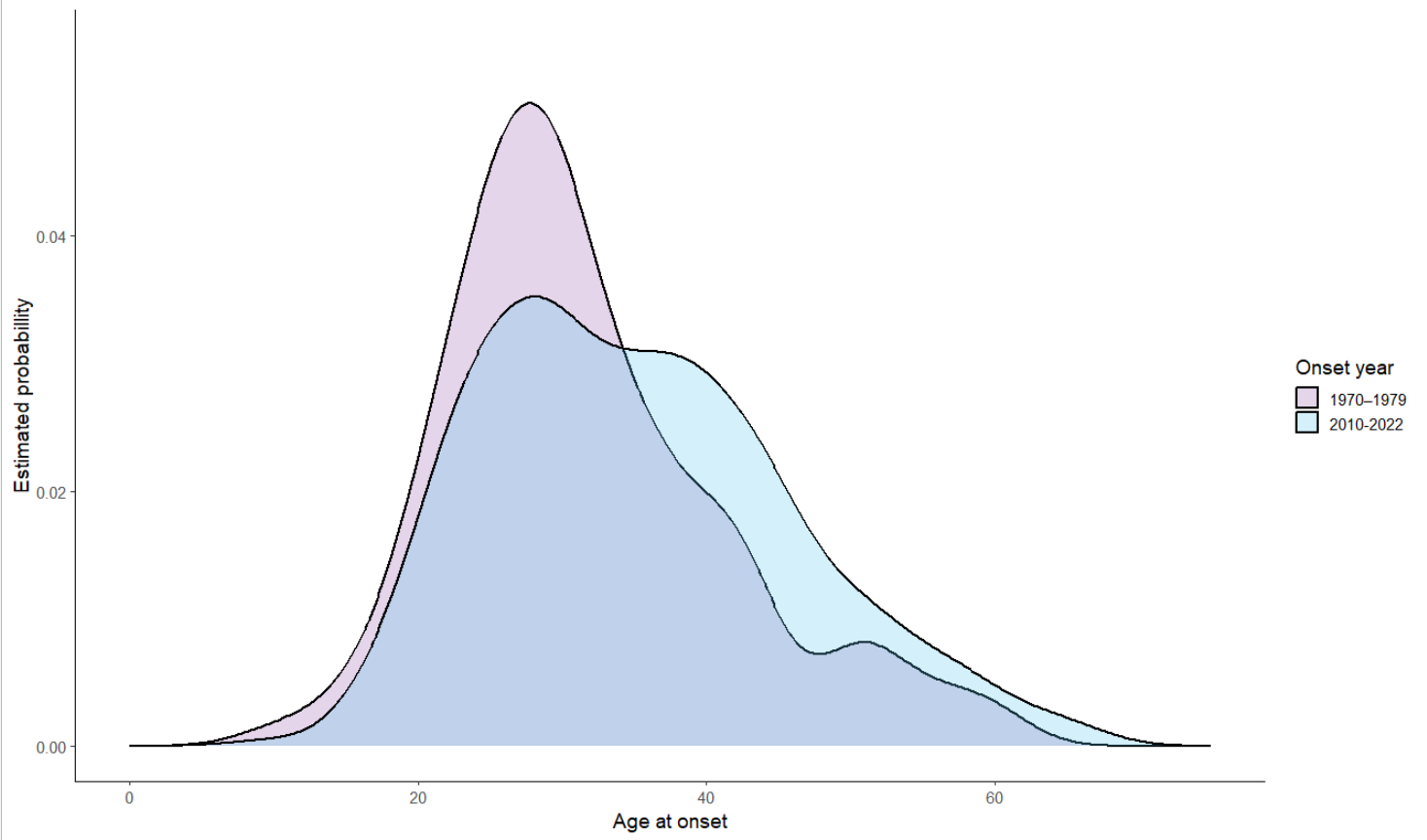


**eFigure 5**. Distribution of age at onset of relapsing-remitting multiples sclerosis during 1970-1979 compared to 2010-2022, women only.
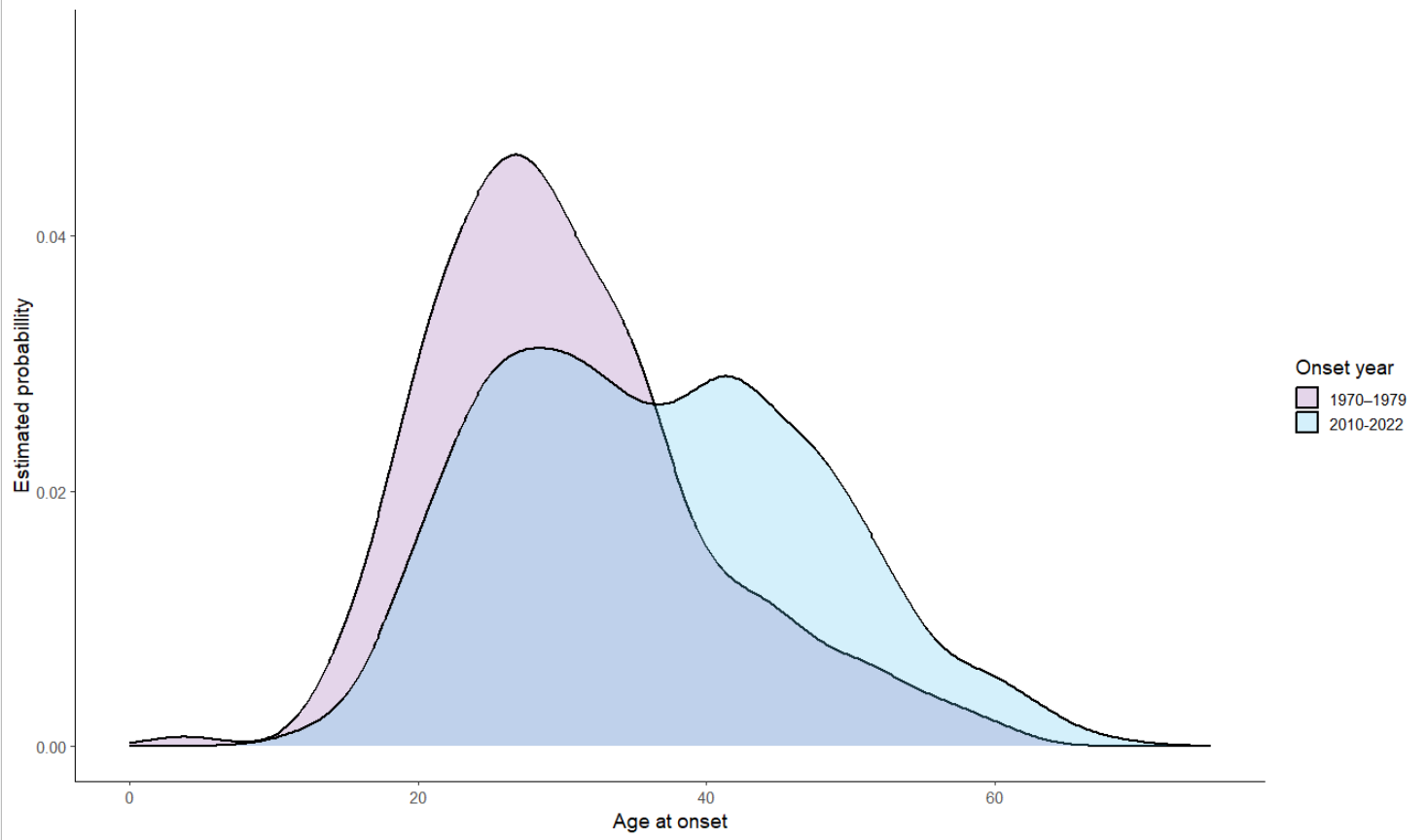

Supplement: Supplementary file 1 — Supplementary file1 (DOCX 602 KB) [file 415_2023_12047_MOESM1_ESM.docx]
